# New record of *Amblyomma dissimile* in *Caiman yacare* in Southernmost Brazil: emerging risks of animal translocation

**DOI:** 10.1590/S1984-29612026031

**Published:** 2026-07-31

**Authors:** Clara Murussi Beheregaray, Luiza Rodrigues Tirelli, Laura Berger Nunes, Vinícius Baggio-Souza, Jorge da Silva Lima, David Driemeier, Saulo Petinatti Pavarini, João Fabio Soares

**Affiliations:** 1 Universidade Federal do Rio Grande do Sul – UFRGS, Faculdade de Veterinária, Departamento de Patologia Clínica Veterinária, Laboratório de Protozoologia e Rickettsioses Vetoriais, Porto Alegre, RS, Brasil; 2 GramadoZoo, Gramado, RS, Brasil; 3 Universidade Federal do Rio Grande do Sul – UFRGS, Faculdade de Veterinária, Departamento de Patologia Clínica Veterinária, Setor de Patologia Veterinária, Porto Alegre, RS, Brasil

**Keywords:** Pantanal alligator, tick, Pantanal, Rio Grande do Sul, Jacaré-do-pantanal, carrapato, Pantanal, Rio Grande do Sul

## Abstract

In Brazil, the species *Amblyomma dissimile* occurs mainly in the North and Midwestern regions and does not naturally occur in the Southern region. Here, we report the second introduction of this tick in Rio Grande do Sul, resulting from the translocation of its host, *Caiman yacare* (pantanal alligator), from the state of Mato Grosso to Gramado (RS), which highlights the risks associated with transporting wild animals between regions with different climates.

## Resumo

No Brasil, a espécie *Amblyomma dissimile* ocorre majoritariamente nas regiões norte e centro-oeste, não ocorrendo naturalmente na região sul do país. Registramos a segunda introdução desse carrapato no estado do Rio Grande do Sul, proveniente de uma translocação do seu hospedeiro, *Caiman yacare* (jacaré-do-pantanal), vindo do estado do Mato Grosso a Gramado (RS), fato que alerta sobre os riscos oriundos dos transportes de animais silvestres entre regiões de climas e biomas distintos.

## Introduction

The introduction of exotic species in different ecosystems is a concern that has grown worldwide as travel and transportation is facilitated. These exotic species may include a plethora of parasites, as their mostly reduced size and disguise mechanisms make them extremely inconspicuous hitchhikers.

There are ample reports of this occurrence, especially with humans and domestic animals. Recent cases in human hosts in Brazil include *Dermacentor andersoni* Stiles, 1908 and *Dermacentor variabilis* (Say, 1821), from travelers returning to the country from the United States (Faccini-Martínez et al*.*, 2021; Martins & Pinter, 2022), and *Amblyomma coelebs* Neumann, 1899 from a traveler returning from the Amazon to Rio Grande do Sul (Souza et al., 2022), where these tick species do not naturally occur. Other parasites can be introduced in domestic animal hosts, such as *Hyalomma marginatum* Koch, 1844 on horses from Portugal (Labruna et al*.*, 2001), the already well-established species *Rhipicephalus sanguineus sensu lato*, originally parasitizing canids in the old world, and *Rhipicephalus microplus* (Canestrini, 1888), which originated from India, parasitizing cattle (Evans et al., 2000; Moudgil et al., 2023).

As anthropic interference with wildlife increases, so does translocation of these animals and their respective parasites. Such was the case with the importation of a white rhinoceros *Ceratotherium simum* (Burchell, 1817) from South Africa to Brazil, which was parasitized by the exotic botfly *Gyrostigma rhinocerontis* (Owen, 1830) (Pachaly et al*.*, 2016).

With the aim of highlighting the importance of acarological surveillance in translocated animals, this work seeks to record the introduction of *A. dissimile* (Acari: Ixodida) into the state of Rio Grande do Sul.

*Amblyomma dissimile* is a hard tick that occurs naturally in the North and Midwestern regions of Brazil (Aragão, 1936), with geographical distribution limited to tropical climate conditions (Polo et al*.*, 2021). Reptiles and amphibians are the most common hosts, harboring larvae, nymphs and adult stages (Guglielmone & Nava, 2010), with a few reports of immature stages found infesting birds (order Ciconiiformes) and mammals (orders Artiodactyla, Carnivora, Didelphimorphia, Primates and Rodentia) (Guglielmone & Nava, 2010; Nava et al., 2017). There are reports in crocodilians such as *Caiman yacare* Daudin, 1802, *Paleosuchus trigonatus* (Schneider, 1801), *Paleosuchus palpebrosus* (Cuvier, 1807), *Caiman crocodilus* Linnaeus, 1758 and *Crocodylus moreletii* (A.H.A. Duméril and Bibron, 1851) (Nava et al., 2017; Witter et al*.*, 2016). There are sparse autochthonous reports in the Northeast (Bassini-Silva et al., 2024) in lizards of the genus *Tropidurus, Ameiva*, and *Iguana iguana* (Linnaeus, 1758) (Dantas-Torres et al., 2008). In the Southern region, *A. dissimile* infestation was reported in a captive green anaconda *Eunectes murinus* (Linnaeus, 1758) translocated from the Amazon (Brum & Rickes, 2003). More species of hard ticks from *Amblyomma* genus can be found in five of the six crocodilians that occur in Brazil, those are *Amblyomma fuscum* Neumann, 1907*, Amblyomma geayi* Neumann, 1899*, Amblyomma humerale* Koch, 1844, and *Amblyomma rotundatum* Koch, 1844 (Martins & Acosta, 2021).

Under experimental conditions, *A. dissimile* is a vector of the bacteria *Ehrlichia ruminantium*, which causes fatal heartwater in ungulates (Kasari et al., 2010), although very unlikely under natural conditions. Furthermore, *E. ruminantium* has not yet been reported in Brazil (CFSPH, 2024). There are also reports of molecular detection of *Rickettsia bellii*, ‘*Candidatus* Rickettsia colombianensi’, *Anaplasma* spp*.* and *Hepatozoon* spp. in *A. dissimile* found on Northern jararaca *Bothrops atrox* (Linnaeus, 1758), in the state of Pará (Ogrzewalska et al., 2019).

Rio Grande do Sul is the southernmost state in Brazil. Due to its unique subtropical climate and distinct vegetation, such as that of the Pampa biome, its ixodofauna differs from any other Brazilian region (Berger et al., 2024).

## Material and Methods

**Figure 1:**
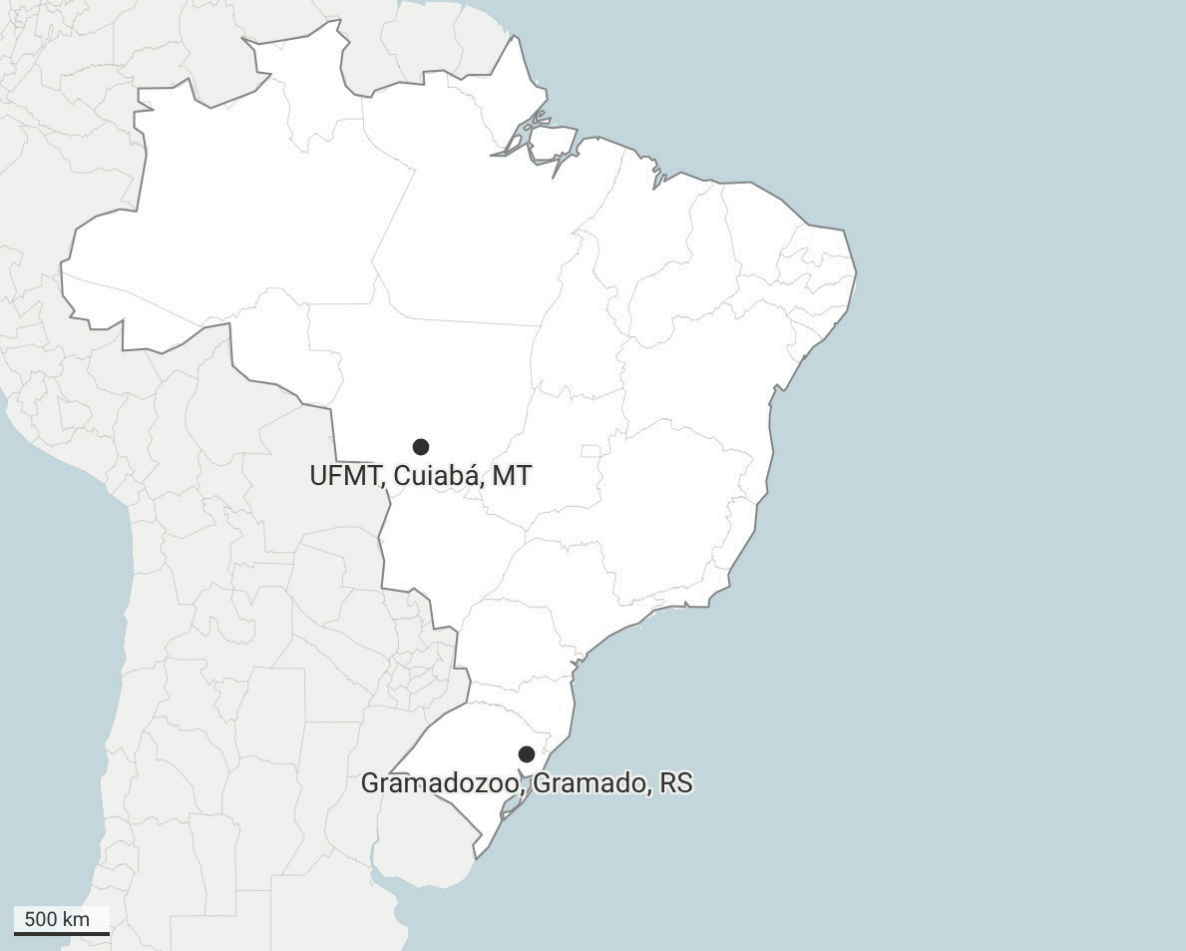
Geographic area of Brazil. Pointing out Geographical locations of UFMT in the state of Mato Grosso (MT) and Gramado Zoo in the state of Rio Grande do Sul (RS).

In May 2021, seven *C. yacare* were translocated from the Federal University of Mato Grosso Zoo, in Cuiabá municipality, state of Mato Grosso, Midwestern region, to Gramado Zoo, in Gramado, Rio Grande do Sul, Southern region, as the Federal University of Mato Grosso Zoo closed down. The distance travelled is estimated to be 1470 km (Figure 1). All individuals were captive-born adults. These animals died in the subsequent month and were sent to necropsy in Setor de Patologia Veterinária of the Universidade Federal do Rio Grande do Sul. The cause of death is presumed to be stress-related, as the animals presented different lesions such as bacterial enteritis, fungal pneumonia and erosive gastric lesions. Ectoparasites were collected and stored in 70% ethanol for morphological identification according to dichotomous keys for adult ticks of the *Amblyomma* genus (Martins et al*.*, 2024).

## Results

**Figure 2:**
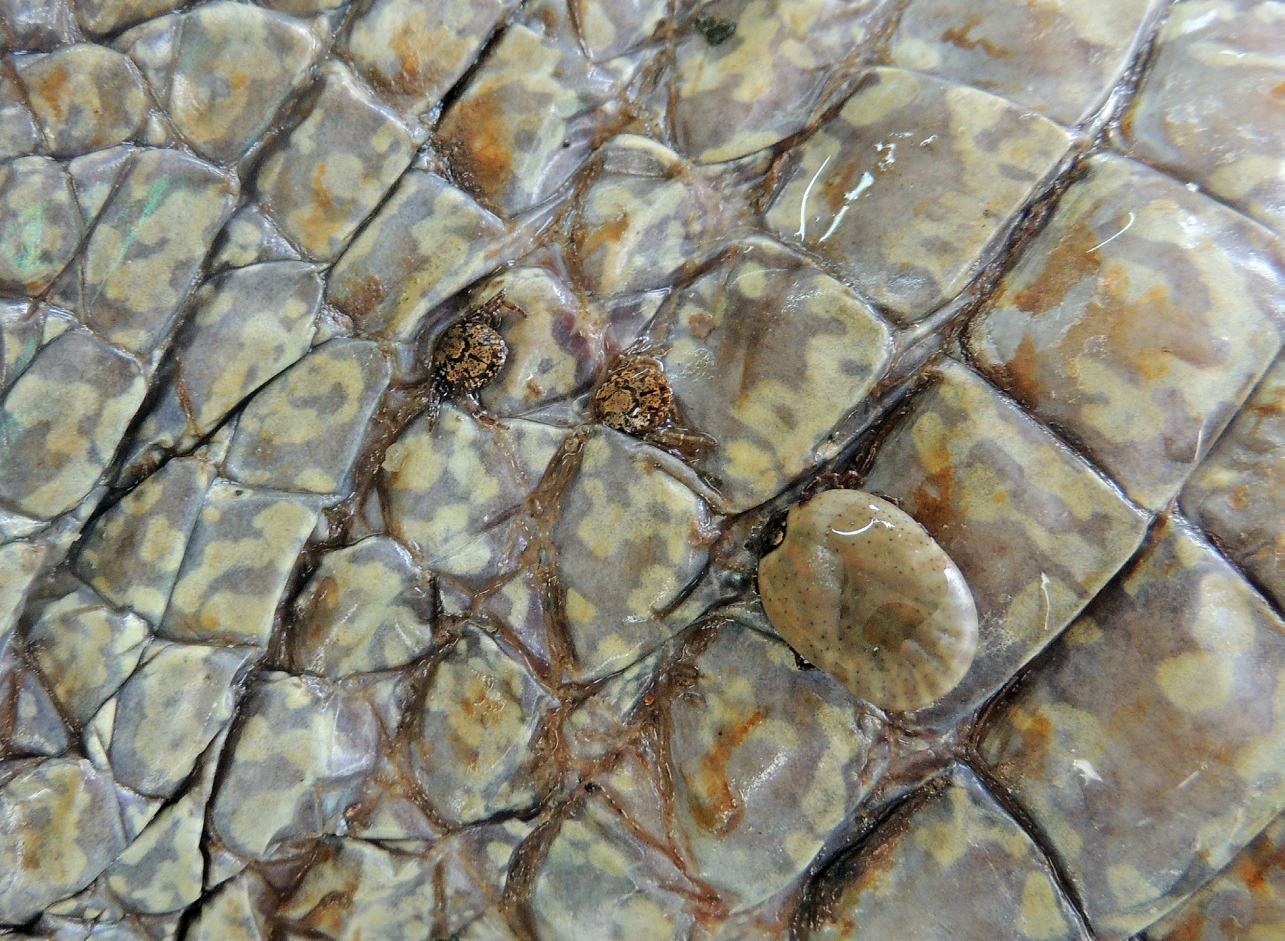
*Amblyomma dissimile*, two males and one female, on a *Caiman yacare* skin, neck and torso, dorsal region.

Ticks were found on two (2/7; 28.6%) animals (Figure 2). The ticks were morphologically identified as *A. dissimile*. In total, five females, nine males (4 females and three male on one *Caiman yacare*, one female and six males on the other) were collected from the individuals.

The ticks found are deposited by numbers #2592 #2781 in the “Coleção de Vetores do Laboratório de Protozoologia e Rickettsioses Vetoriais”.

## Discussion

*Amblyomma dissimile* is an exotic tick in the Southernmost Region of Brazil. The introduction of such ticks, if they are able to adapt to the change of environment, poses a sanitary threat to the state of Rio Grande do Sul, such as new host-vector relations with native species and spread of exotic pathogens. A similar occurrence was reported in 2003 involving an *Eunectes murinus* (Linnaeus, 1758) translocated from a Zoo located in the Amazon region to a Zoo in Rio Grande do Sul (Brum & Rickes, 2003), and we now report a second occurrence after 22 years. The same authors had already warned of the threat of introducing exotic animals to a region without following proper sanitary and disinfection protocols, in a report of importation of a white rhinoceros from South Africa to the Zoológico de Sapucaia do Sul, Rio Grande do Sul, when it was observed parasitism by *Gyrostigma rhinocerontis,* an exotic species of botfly (Brum et al*.*, 1996), which also occurred in the state of São Paulo (Pachaly et al*.*, 2016).

The same tick (*A. dissimile*) was also reported in the states of São Paulo and Paraná, although it is much more probably another case of introduction of animals transported from other regions of Brazil where the tick occurs naturally, considering that the reports were in captive animals and there is no evidence of the species establishment in those states and climates (Martins et al., 2024).

Not only are the ticks and other ectoparasites a threat to the local ecosystem, but they may also act as vectors for other exotic pathogens. Such was the case in 2019, when a human infested with *A. coelebs* ticks in the Amazon Rainforest traveled to the state of Rio Grande do Sul, and both the ticks and the human tested positive for *Rickettsia amblyomattis* (Souza et al*.*, 2022), which has a single reported occurrence in the state (Krawczak et al., 2016).

The southernmost region’s climate conditions are currently incompatible with some tick species’ ecology, mainly due to temperature, which is colder than the tick’s preferences, along with sparser shrubs and tree formations. These factors may be altered, however, given expected environmental and climatic changes forced by anthropological pressure (Polo et al*.*, 2021).

As we recognize that fast globalization and the heavy flow of human travel across borders are an impending risk for the introduction of new populations of parasites to countries or regions, the situation is even more worrisome when it comes to animal translocation and the enormous variety of parasites associated with each species. Therefore, it is imperative that sanitary actions, alongside rigorous acarological surveillance, are properly established and adhered to when it comes to animal translocation, to minimize accidental transportation and consequent introduction and possible establishment of new vector species in regions previously free of that specific parasite.

## Data Availability

The data supporting this article’s main conclusions are included in the article.
